# Supplementary material for: Perspectives and Factors Affecting the Preventive Behavior Pertinent to COVID-19 among School Employees in Chiang Mai, Thailand: A Cross-Sectional Study
Source: Int J Environ Res Public Health. 2022 May 6;19(9):5662. doi: 10.3390/ijerph19095662 (PMC9104893; doi:10.3390/ijerph19095662)
Supplement: Supplementary file 1 [file ijerph-19-05662-s001.zip › ijerph-1667557-supplementary.pdf]

Table S1. The reliability test of the questions for assessing school employees' agreement and actions in accordance with the preventive measures.

Questions	Cronbach's Alpha	
	**Agreement (Agree/Not agree)**	**Actions (Consistently/Partially/ Not performed)**
1. You must follow the information on the epidemic situation from reliable sources.	0.8101	0.8934
2. You must observe your own abnormal symptoms. If there are any respiratory symptoms, you should stop working and consult a medical team immediately.	0.8114	0.8883
3. You must strictly follow preventive measures such as washing your hands often, wearing a cloth mask or a mask and keeping distance between people and avoid going to crowded places.	0.8180	0.8872
4. You must inform parents of supervising students bringing their own personal items and protective equipment to school.	0.8186	0.8735
5. You must communicate knowledge. Advice or provision of public relations materials to prevent and reduce the risk of spreading COVID-19.	0.8074	0.8696
6. You must clean the teaching aids or equipment that is a high-risk touch point after every use.	0.8174	0.8770
7. You must supervise the seating arrangements within the school premises in accordance with the basic principle, the distance between people is at least 1 - 2 meters.	0.8210	0.8730
8. You must inspect, supervise, and follow up on student attendance.	0.8632	0.8781
9. You must perform health screening for everyone who enters the school according to the procedures.	0.8023	0.8741
10. You must observe groups of students with behavioural problems or students who do not cooperate with the measures set by the teacher in order to receive assistance.	0.8175	0.8686
11. You must communicate your knowledge of stress and the stress management process for students and personnel in educational institutions.	0.8101	0.8730
Scale reliability coefficient	0.8347	0.8879

Table S2. The reliability test of the six dimension of school assessment questions

Dimensions	items	Cronbach's Alpha
**Overall**	44	0.8480
**Primary dimension**	20	0.7782
(1) Safety from reducing the spread of disease		
**Secondary dimensions**	24	0.7782
(2) Learning	4	0.7772
(3) Concerned over disadvantaged children	6	0.8196
(4) Benefits and Coverage	5	0.7713
(5) Policy	5	0.7612
(6) Finance Management	4	0.7768

Table S3. Self-Assessment Form for Educational Institutions to prepare before the school is opened to monitor and prevent the spread of Covid-19

****

Academy location______________________

Subdistrict_______________District_____________________Province_________

Telephone_________________

Assessment Criteria

Sequent	Subject	Yes	No	Remark
**1.**	**Safety from reducing the spread of disease**			
1	Is there a screening measurement for students, teachers and visitors before entering the school campus?			
2	Are there measurements to observe the risk of coughing, congested nose, sore throat, unusual fatigue, difficulty breathing, loss of sense of taste or smell and record these results from the students, teachers and visitors before entering the school campus?			
3	Is there a policy for teachers and students to wear cloth or surgical masks before entering school campus?			
4	Does the school prepare cloth or surgical masks for the person asking for it or forget to bring it before entering the school campus?			
5	Is there an adequate hand washing spot/area in the school campus?			
6	Does the school prepare enough hand sanitizer at the entrance, in front of the classrooms and canteens?			
7	Is there a social distancing arrangement of seatings and tables in the classrooms, at the canteens and resting areas of 1-2 meters?			
8	Is there a clear sign to show that there should be social distancing?			
9	In the case that the classroom cannot be set in a social distancing space, did the school set the alternating school days for each classroom grade or divide the number of students accordingly?			

10	Does the school disinfect all classrooms and teaching equipment such as the computer room, music room, sports equipment?			
11	Does the school disinfect common areas of contact that are cleaned daily, such as tables, chairs, handrails, elevators, door bolts, door handles and windows?			
12	Does the trash bin have a lid?			
13	Is there a repair of doors, windows and fans in each classroom for use and has good air circulation?			
14	Is there a diving of a small group of students in the classroom to do the activities?			
15	Is there an adjustment of time to do PR activities after lining up to respect the National flag?			
16	Does the school set aside time for student activities or even lunch break?			
17	Is there social distancing when line up to do activities?			
18	Is there a rule that mentions that you should use your own personal belongings without sharing to others such as glass of water, fork and spoon, toothbrush, toothpaste and handkerchief?			
19	Is there a nurse room or isolation room to quarantine Covid 19 patients from others?			
20	Are there students who are leading health volunteers who volunteer to take care of the health of their fellow students or take care of the Juniors?			

1. Learning

21	Is there a PR sign advising on the practice of hygiene such as a proper and right way to clean your hands, to wear a mask, social distancing and knowledge about Covid 19.			
22	Is there any preparation for teaching management taking into account the learning according to age and in accordance with social, emotional and intellectual development?			
23	Is there a measurement to determine the duration of using online media in educational institutions in (Primary school)l children no more than 1 hour per day and in (Middle school) children no more than 2 hours per day?			

24	Is there a Health knowledge social media being used through Website, Facebook, Line, QR code and email?			

3. Concerned over disadvantaged children

25	A spare mask is being prepared for children or not?			
26	Has the teaching style been adapted to the context of learning in this covid situation?			
27	Are there measurements to encourage students to receive basic health care services?			
28	Are there measurements for cleaning and arranging the environment of the accommodation and sleeping quarters to be hygienic? (There should be a proper daily schedule if there is accommodation or sleeping quarters)			
29	Are there measurements for cleaning and arranging the environment in accordance with religious practice ordinances? (There should be a proper daily schedule in case there is a place to practice religion)			
30	Are there measurements to care for students with developmental learning disabilities or emotional behavioral disabilities, including ADHD and autistic children who are able to attend classes with normal children?			

4. Benefits and Coverage

31	Is there a well prepared plan to support teaching management for students who are sick, quarantined or school closures?			
32	Is there a communication practice to reduce social stigma?			
33	Is there a guideline on stress management for teachers and school personnel?			
34	Is there a risk history check of students and staff, including checking whether the quarantine is completed for 14 days before returning to campus and every time that the school starts?			
35	Is there a guideline for students, teachers, and personnel suspected of being infected with Covid 19 that will not affect his or her leave or school holidays?			

5. Policy

36	Is there effective communication or PR about Covit 19 to the students, teachers, staff and parents by meeting or virtual at least 1 day before the start of school?			
37	Is there a written policy and guideline that will protect the spread of Covid 19 in the school in written contract and with clear evidence?			
38	Is there a School Committee Board Meeting?			
39	Has there been an appointed team to work on the prevention of the epidemic of the Covid 19 with clear and defined roles and responsibilities?			
40	Is there a measurement to manage cleanliness on the school bus, seating social distance in a bus or is there a clear mark on the seating location? (if there is school bus)			

6. Finance Management

41	Are there any necessary and appropriate budget plans for preventing the spread of Covid 19?			
42	Is there an arrangement to purchase any materials or equipment to protect against Covid 19 for students and staff in school such as cloth mask, surgical mask, soap or hand sanitizer.			
43	Is there a coordination in seeking funding sources from agencies, organizations or private sectors such as local companies, department stores or NGO to carry out activities to prevent the spread of Covid19?			
44	Are there additional personnel to take care of students and the environment on the school campus?			

Assessment results

Ranking	Assessment criteria
Green	Passed all 44 items
Yellow	Passed 1-20 all items<br>But failed 13-44 items either one of it
Red	Failed 1-20 items either one of it

Interpretation

- Green color means school is able to open.
- Yellow color means school is able to open but must be improved to meet the required standards.
- Red means school is unable to open and must be improved to meet the required standards and/or re-evaluated.

Name of assessor______________________

Date of assessment_____________________

Remark: Educational Institutions self-assessment via online link
Https:stopcovid.amamai.moph.go.th

must be improved to meet the established standards or re-evaluated

(73)

1. Safety from reducing the spread of germs

1. Is there a screening measurement for students, teachers and visitors before entering the school campus or not?
2. Are there measurements to observe the risk of coughing, congested nose, sore throat, unusual fatigue, difficulty breathing, loss of sense of taste or smell and record these results from the students, teachers and visitors before entering the school campus?
3. Is there a policy for teachers and students to wear cloth or surgical masks before entering school campus?
4. Does the school prepare cloth or surgical masks for those requested for it or forget to bring it before entering the school campus?
5. Is there an adequate hand washing spot/area with soap in the school campus?
6. Does the school prepare enough hand sanitizer at the entrance, in front of the classrooms and canteens?
7. Is there a social distancing arrangement of seatings and tables in the classrooms, at the canteens and resting areas of 1-2 meters?
8. Is there a clear sign or spot to show that there should be social distancing?
9. In the case that the classroom cannot be set in a social distancing space, did the school set the alternating school days for each classroom grade or divide the number of students accordingly?
10. Is there a deep cleaning of all classrooms and teaching equipment (such as computer room, music room, sports equipment)?
11. Is there a deep cleaning of common areas of contact that are cleaned daily, such as tables, chairs, handrails, elevators, door bolts, door handles and windows?
12. Does the trash bin have a lid?
13. Is there an improvement or repair of doors, windows and fans in each classroom for use and has good air circulation? (74)
14. Is there a diving of a small group of students in the classroom to do the activities?
15. Is there an adjustment of time to do PR activities after lining up to respect the national flag?
16. Does the school set aside time for student activities or even lunch break?
17. Is there social distancing when line up to do activities?
18. Is there a rule that you should use your own personal belongings without sharing to others such as glass of water, fork and spoon, toothbrush, toothpaste and handkerchief?
19. Is there a nurse room or isolation room to separate covid 19 patients from others?

20. Are there students who are leading health volunteers who volunteer to take care of the health of their fellow students or take care of the Juniors?

2. Learning

21. Is there a PR sign advising on the practice of hygiene (such as a proper and right way to clean your hands, to wear a mask, social distancing and knowledge about Covid 19).
22. Is there any preparation for teaching management taking into account the learning according to age and in accordance with social, emotional and intellectual development?
23. Is there a measurement to determine the duration of using online media in educational institutions in (primary school)l children no more than 1 hour per day and in (middle school) children no more than 2 hours?
24. Health knowledge social media is used through Website, Facebook, Line, QR code and email channels or not?

3. **Concerned over disadvantaged children**

25. A spare mask is prepared for children or not?
26. Has the teaching style been adapted to the context of learning in this covid situation?
27. Are there measurements to encourage students to receive basic health care services?
28. Are there measurements for cleaning and arranging the environment of the accommodation and sleeping quarters to be hygienic? (Proper daily schedule if there is accommodation or sleeping quarters)
29. Are there Measurements for cleaning and arranging the environment in accordance with religious practice ordinances? (Proper daily schedule in case there is a place to practice religion) (75)
30. Are there measurements to care for students with developmental learning disabilities or emotional behavioral disabilities, including ADHD and autistic children who are able to attend classes with normal children?

4. Benefit and coverage

31. Is there a well prepared plan to support teaching management for students who are sick, quarantined or school closures?
32. Is there a communication practice to reduce social stigma?
33. Is there a guideline on stress management for teachers and school personnel?
34. Is there a risk history check of students and staff, including checking whether the quarantine is completed for 14 days before returning to campus and every time that the school starts?
35. Is there a guideline for students, teachers, and personnel suspected of being infected with Covid 19 that will not affect his or her leave or school holidays?

5. Policy

36. Is there effective communication or PR about Covit 19 to the students, teachers, staff and parents by meeting or virtual at least 1 day before the start of school?
37. Is there a written policy and guideline that will protect the spread of Covid 19 of the school in written and with clear evidence?
38. Is there a School Committee Board Meeting?
39. Has there been an appointed team to work on the prevention of the epidemic of the Covid 19 with clear and defined roles and responsibilities?
40. Is there a measurement to manage cleanliness on the school bus, seating social distance in a bus or is there a clear mark on the seating location? (if there is school bus)

6. Finance Management

41. Are there any necessary and appropriate budget plans for preventing the spread of covid 19?
42. Is there an arrangement to purchase any materials or equipment to protect against covid 19 for students and staff in school such as cloth mask, surgical mask, soap or hand sanitizer.
43. Is there a coordination in seeking funding sources from agencies, organizations or private sectors such as local companies, department stores or NGO to carry out activities to prevent the spread of covid19?
44. Are there additional personnel to take care of students and the environment on the school campus?
